# Revuforj (revumenib): a novel menin inhibitor for relapsed or refractory acute leukemia

**DOI:** 10.1097/MS9.0000000000004662

**Published:** 2026-01-06

**Authors:** Abdullah Imtiaz, Ayesha Nazeer, Ehtisham Haider, Sakan Binte Imran

**Affiliations:** a Department of Medicine, Wah Medical College, Wah Cantt, Rawalpindi, Pakistan; b Department of Medicine, Punjab medical college, Faisalabad, Pakistan; c Department of Medicine, Sir Salimullah Medical College, Bangladesh

*To the Editor,*

Acute leukemias, including acute myeloid leukemia and acute lymphoblastic leukemia, are aggressive, rapidly proliferative, hematologic malignancies that induce different phenotypic, genetic, and molecular mutations^[1]^. The multiplicity in chromosomal aberration leads to bone marrow failure, resulting in anemia, infections, and bleeding tendencies. The poor disease outcomes, despite chemotherapy and advanced therapeutic targets, expedite the relapsed/refractory (r/r) cases that are difficult to cope. Revumenib is an innovative targeted treatment with promising activity in r/r cases of acute leukemias with lysin methyltransferase 2A (KMT2A) rearrangement. The interaction between the KMT2A and the scaffolding protein, menin, yields leukemogenic transcription, which is blocked by revumenib. Preclinical studies show revumenib also induces apoptosis and cell cycle arrest in leukemic stem cells^[2]^. It promotes the differentiation and exerts anti-leukemic activity^[3]^. Revumenib paves the way for improved clinical outcomes, overall survival, and complete remission with partial hematologic recovery (CRh). A US budget-impact analysis projects an incremental 3-year cost of USD 58 million per 1-million-member health plan; cost-effectiveness is accepted only at willingness-to-pay thresholds ≥ USD 200 000 per Quality-Adjusted Life Year (QALY)^[4]^. The FDA approval of Revuforj has introduced a targeted dimension to treat r/r cases of acute leukemia, bringing optimistic hope for this challenging patient population.

Recently, the usage of the first-of-its-kind oral menin inhibitor Revuforj (revumenib) was approved, which became a significant breakthrough in the management of relapsing or resistant acute leukemia that has KMT2A rearrangements. Revumenib functions by disrupting the interaction between menin and KMT2A fusion proteins, thus resulting in the inhibition of aberrant HOX/MEIS transcriptional programs and differentiating leukemic cells^[5]^. This is a direct response to the leukemogenic defect in KMT2A-rearranged malignancies, a transition of the traditional cytotoxic basis to precision molecular. Revumenib also has a combined CR and CRh rate of 22% (95% CI: 12.735.8) in heavily pretreated patients in a pivotal phase I/II AUGMENT-101 trial (NCT04065399), with a median time to response of 1.9 months^[2]^. The clinical relevance of these remissions is so important that revumenib was approved by the U.S. Food and Drug Administration in November 2024 with relapsed or refractory acute leukemia having KMT2A translocation. Differentiation syndrome (21 %, including two fatal cases), QTc prolongation >500 ms (8 %, one Torsades de Pointes), cytopenias, and infections were the most common adverse events; dexamethasone prophylaxis and ECG monitoring are mandated^[6]^. Revumenib can be considered an important development in the treatment of KMT2A-rearranged leukemia, and its further use as a part of frontline and combination therapy in NPM1-mutated and NUP98-rearranged leukemia is under investigation^[2]^.

The introduction of Revuforj (revumenib), a new drug by the FDA, can be regarded as a paradigm shift in the therapeutic process of the relapsed or refractory acute leukemia with KMT2A rearrangements, as it is the introduction of precision medicine into the difficult environment. Revumenib selectively inhibits the menin–KMT2A interaction to reestablish normal differentiation programs and provides a significant clinical benefit in locations where traditional therapy is mostly unsuccessful. Although treatment-related toxicity is still essential to pay attention to, the remission rates and manageable safety profile of it allow recognizing its potential transformative nature. Furthermore* analysis of frontline and combination therapies, such as NPM1-mutated and NUP98-rearranged leukemia, will continue to establish its place in enhancing long-term outcomes and develop personalized. This manuscript complies with TITAN Guidelines, 2025, declaring no use of AI^[7]^.
